# Identification and characterization of unknown disturbances in a structured population using high-throughput phenotyping data and measurement of robustness: application to growing pigs

**DOI:** 10.1093/jas/skae059

**Published:** 2024-03-05

**Authors:** Vincent Le, Tom Rohmer, Ingrid David

**Affiliations:** GenPhySE, Université de Toulouse, INRAE, ENVT, F-31326 Castanet Tolosan, France; Alliance R&D, 35650 Le Rheu, France; GenPhySE, Université de Toulouse, INRAE, ENVT, F-31326 Castanet Tolosan, France; GenPhySE, Université de Toulouse, INRAE, ENVT, F-31326 Castanet Tolosan, France

**Keywords:** disturbances detection, longitudinal phenotype, pig, robustness

## Abstract

Improving the robustness of animals has become a priority in breeding due to climate change, new societal demands, and the agroecological transition. Components of animal robustness can be extracted from the analysis of the adaptive response of an animal to disturbance using longitudinal data. Nonetheless, this response is a function of animal robustness as well as of disturbance characteristics (intensity and duration). To correctly assess an animal’s robustness potential, it is therefore useful to know the characteristics of the disturbances it faces. The UpDown method, which detects and characterizes unknown disturbances at different levels of organization of the population (e.g., individual, pen, and batch disturbances), has been proposed for this purpose. Furthermore, using the outputs of the method, it is possible to extract proxies of the robustness of animals. In this context, the objective of the study was to evaluate the performances of the UpDown method to detect and characterize disturbances and quantify the robustness of animals in a genetic framework using different sets of simulations, and to apply this method to real pig longitudinal data recorded during the fattening period (body weight, cumulative feed intake, and feeding rate). Based on the simulations, the specificity of the UpDown method was high (>0.95). Its sensitivity increased with the level of organization exposed (from 0.23 to 0.32 for individual disturbances, from 0.45 to 0.59 for pen disturbances, and from 0.77 to 0.88 for batch disturbances). The UpDown method also showed a good ability to characterize detected disturbances. The average time interval between the estimated and true start date or duration of the disturbance was lower than 3 d. The correlation between the true and estimated intensity of the disturbance increased with the hierarchical level of organization (on average, 0.41, 0.78, and 0.83 for individual, pen, and batch disturbance, respectively). The accuracy of the estimated breeding values of the proxies for robustness extracted from the analysis of individual trajectories over time were moderate (lower than 0.33). Applied to real data, the UpDown method detected different disturbances depending on the phenotype analyzed. The heritability of the proxies of robustness were low to moderate (ranging from 0.11 to 0.20).

## Teaser

The UpDown method is an efficient new tool to detect and characterize unknown disturbances using high-throughput phenotyping data. It also makes it possible to obtain new measurements of animal robustness.

## Introduction

Due to climate change that will inevitably lead to more extreme events (Pasqui and Di Giuseppe, 2019), new societal demands that imply changes in breeding systems (Alonso et al., 2020), and the agroecological transition associated with greater feed variability (Phocas et al., 2014), livestock will undergo more and more disturbances of various origins. In this context, improving the robustness of animals is defined as “the ability to combine a high production potential with resilience to stressors, allowing for the unproblematic expression of a high production potential in a wide variety of environmental conditions” (Knap, 2005), has become a priority in breeding. Two options for breeding for animal robustness are considered in the literature (Rauw and Gomez-Raya, 2015). The first one consists of directly including measurable robustness traits in the breeding objective. However, robustness is a complex trait that is difficult to phenotype (Friggens et al., 2017). Today, traits included in breeding goals that may be associated with robustness are mainly related to the health status of animals during a specific period or to the longevity of reproductive animals (Knap, 2005; Berghof et al., 2019; Knap and Doeschl-Wilson, 2020; Lenoir et al., 2022). The second option consists of estimating the environmental sensitivity of animals by modeling and integrating the corresponding genetic value into the selection objective. The most commonly used model for this is the reaction norm model, which necessitates the environment to be described in the form of a gradient (Falconer, 1990; Kolmodin et al., 2002; Calus et al., 2004). More sophisticated models have recently been proposed, using high-throughput phenotyping data to study the dynamic nature of animals’ adaptive responses to disturbance (Codrea et al., 2011; Sadoul et al., 2015; Revilla et al., 2019; Nguyen-Ba et al., 2020; Poppe et al., 2020). Some of these methods require the disturbance to be identified (e.g., weaning stress), while others do not but assume that animals reared together undergo the same disturbance, which makes their response to the disturbance comparable. However, disturbance can occur at different levels of a farm’s organization and does not necessarily affect all contemporary animals. For example, based on the structure of a pig farm where animals are raised in small intra-pen groups and intra-batch groups of pens, an environmental disturbance such as a heat wave will affect all the animals in the batch, whereas a problem with the electronic feeder will only affect the individuals in the pen concerned, and a metabolic disease, for example, will only affect the animal concerned. To the best of our knowledge, very few studies have simultaneously used information from a group of contemporary animals to detect and characterize unknown disturbances, which should nevertheless enable us to gain detection power. Garcia-Baccino et al. (2021) proposed to detect disturbances by analyzing the daily phenotypic variance of a group of animals. Its distribution is considered as a mixture of two normal distributions (disturbed/non-disturbed days). The authors proposed using the probability of belonging to the disturbed distribution for each day as a measure of the environmental challenge that is then included as a covariate in a reaction norm animal model to estimate its environmental sensitivity. Another method to detect disturbances based on the analysis of longitudinal data at the group level was recently proposed, applicable using the “UpDown” package (David et al., 2023) in R software (R Core Team, 2021). The method analyzes the dynamics of longitudinal phenotypes at different group levels, once again using a mixture of two normal distributions to classify elements at each level of organization as disturbed or not and to characterize the detected disturbances. The aim of our study was to evaluate the performance of the UpDown method in depth via simulation in order to detect and characterize disturbances in a population structured into different group levels, and to quantify the robustness of animals in a genetic framework. This method was applied to real body weight (BW), cumulative feed intake (CFI), and the feeding rate longitudinal records of 5,872 pigs in order to perform a genetic analysis of their robustness.

## Materials and Methods

Data were collected in accordance with the applicable national regulations on livestock welfare in France.

### The UpDown method

**Figure 1.**
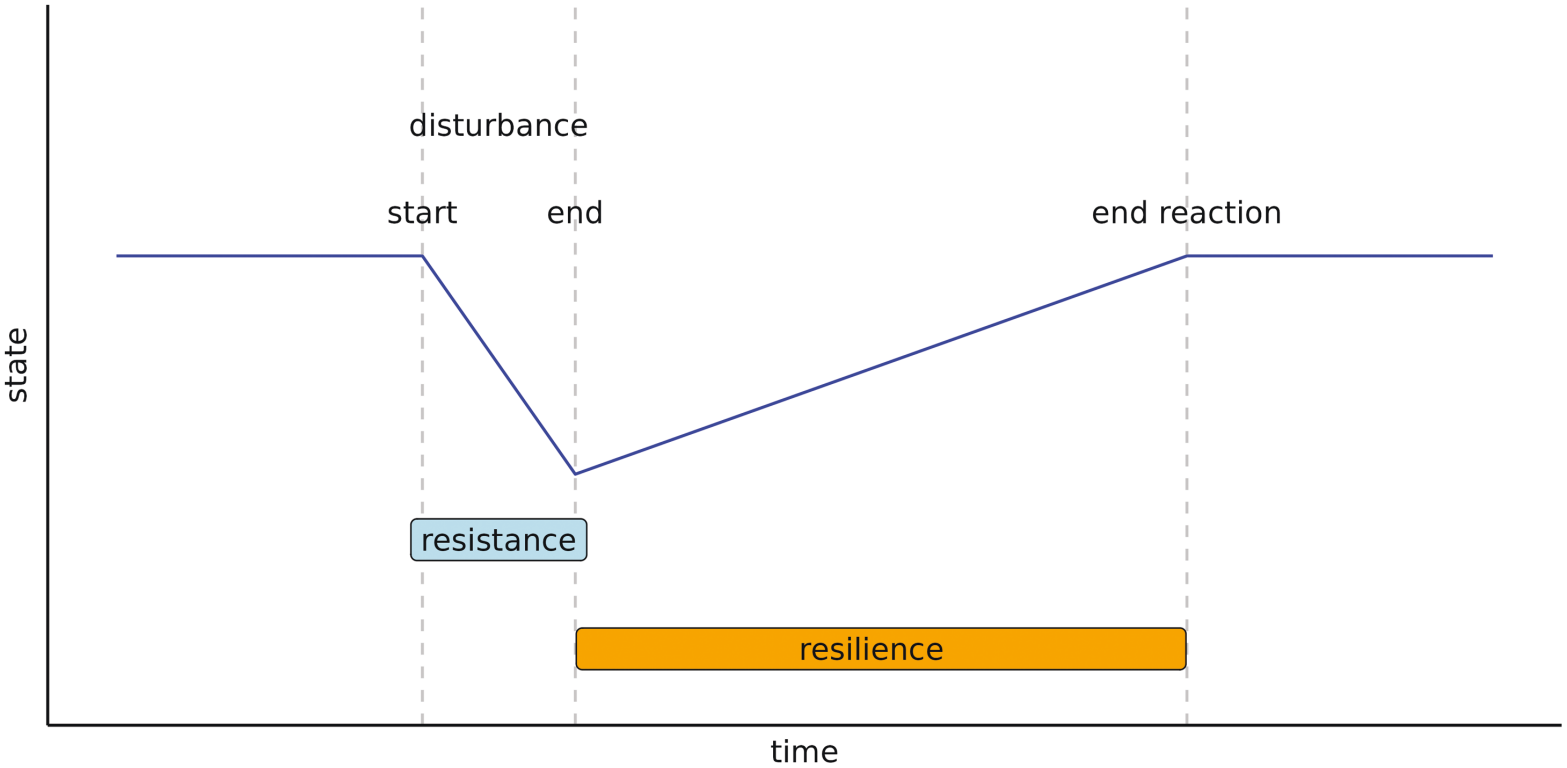
Theoretical elastic changes in the state of an animal facing a disturbance. When the disturbance appears (start line), the importance of change in state will depend on the resistance of the animal and the intensity of the disturbance. As soon as the animal has quit its initial state, the resilience capacity is involved in order to limit the effect of the perturbing factor so that it can return to its initial state.

Let us consider a population organized into different grouping levels of contemporary animals. For example, in the pig farming system, the population is organized into three group levels: individual, pen, and batch. Each batch is made up of a group of several pens and each pen is made up of a group of several animals. In other words, the individuals are included in pens that are included in batches. The UpDown method (David et al., 2023) involves studying longitudinal observations at different group scales to facilitate the detection and characterization of disturbances on the basis of changes in the dynamics of observations over time. Indeed, Sauvant and Martin (2010) consider that the response of animals facing a disturbance can be broken down into two parts: change in state when exposed to the disturbance (part 1), and once the disturbance is over, the animal’s return to its initial state (if it is elastic, part 2). A schema describing changes in state for an elastic system is provided in Figure 1. This dynamic response of an animal to a disturbance can be described in terms of resistance and resilience, which are defined as the capacity of an animal to minimize the impacts of perturbing factors and to quickly return to the pre-perturbed condition (Sadoul et al., 2015; Nguyen-Ba et al., 2020). When the disturbance appears, the importance of change in state will depend on the intensity of the disturbance, the resistance of the animal, as well as on its resilience as soon as the animal has quit its initial state to limit the effect of the perturbing factor. When the disturbance is over, only the resilience capacity is involved until the initial state is restored. The UpDown method consists of two steps: (1) the Up-step identifies the elements facing disturbance at each group level, starting at the individual level and ending at the highest organizational level (i.e., the batch in the pig system); and (2) the Down-step validates elements detected in the Up-step, from the highest to the individual level of organization, and characterizes the detected disturbances (i.e., start date, end date, and intensity). Individual phenotypes are first corrected for fixed effects and for the general trend of the population over time (i.e., the age for growth phenotypes, the number of days in lactation for milk yield, etc.) and smoothed using a Nadaraya-Watson’s kernel regression (Nadaraya, 1964; Watson, 1964). Then, considering that animals have an elastic or plastic response (Sauvant and Martin, 2010) to the disturbance that always goes in the same direction (slowing down or accelerating evolutionary dynamics over time), the Up-step identifies elements subject to at least one disturbance at the different scales by fitting, using an EM algorithm, a mixture of two Gaussian distributions to the minimum slopes of the smoothed trajectories. For levels higher than the individual level, the trajectory was obtained by taking the median value of the sub-level organization at each time point of observation (e.g., the trajectory for a given pen is obtained by taking the median of the observations of the animals in the pen at each different time). The Down-step is carried out in two stages. Firstly, it identifies all the disturbances for each element identified as having undergone at least one disturbance in the Up-step. All dates for which the local minimums of the slope are below the threshold value of the mixture model used in the Up-step are considered as disturbance start dates. The hierarchical level at which the disturbance occurs is validated if a high proportion of the sub-elements making up this hierarchical level have a close disturbance start date (obtained by clustering). The default value of this proportion is 50% in the UpDown R package. Once the hierarchical level of each disturbance has been validated, the program completes the characterization of each disturbance by identifying its end date (date of the first local minimum of the phenotype after the start of the disturbance) and its intensity (slope of the phenotype between the start and end of the disturbance). The outputs of the UpDown package are thus a list of disturbances with their characteristics: hierarchical level affected, disturbance start and end dates, and intensity.

In addition, using the results of the UpDown package, it is possible to evaluate the individual response of each animal to the various disturbances it has undergone at the group level. We propose three statistics for this, derived from the study of the evolutionary dynamics of the smoothed corrected individual phenotype: (1) Smin, the minimum of its first derivative during disturbance exposure (i.e., between the start and the end of the disturbance or end of the observation period if the end of the disturbance is later). The latter should correspond to the primo-response of the animal exposed to a disturbance and, thus, its resistance. The greater Smin is, the greater the resistance will be; (2) Sresil, the slope between the end of the disturbance and the end of the animal’s reaction (date of a local maximum of the slope of the smoothing curve after the end of the disturbance). It should correspond to the ability of the animal to recover after an exposure to a disturbance (e.g., its resilience; the greater Sresil is, the greater the resilience will be); (3) ABT, the area-between the smoothed and theoretical (non-disturbed) trajectories (obtained by linear regression) between the start and the end of the reaction dates that should measure the global reaction of the animal, thus, its robustness. The greater ABT is, the lower the robustness will be. If the end of the reaction is not identified for an animal, neither ABT nor Sresil are computed. It should be noted that for disturbances identified at the individual level, the individual dynamic is used to characterize the disturbance, but this dynamic is also a function of the resistance and resilience of the animal. Thus, the measure of intensity is not valid for this level.

## Materials and Methods

### Simulated data

**Figure 2.**
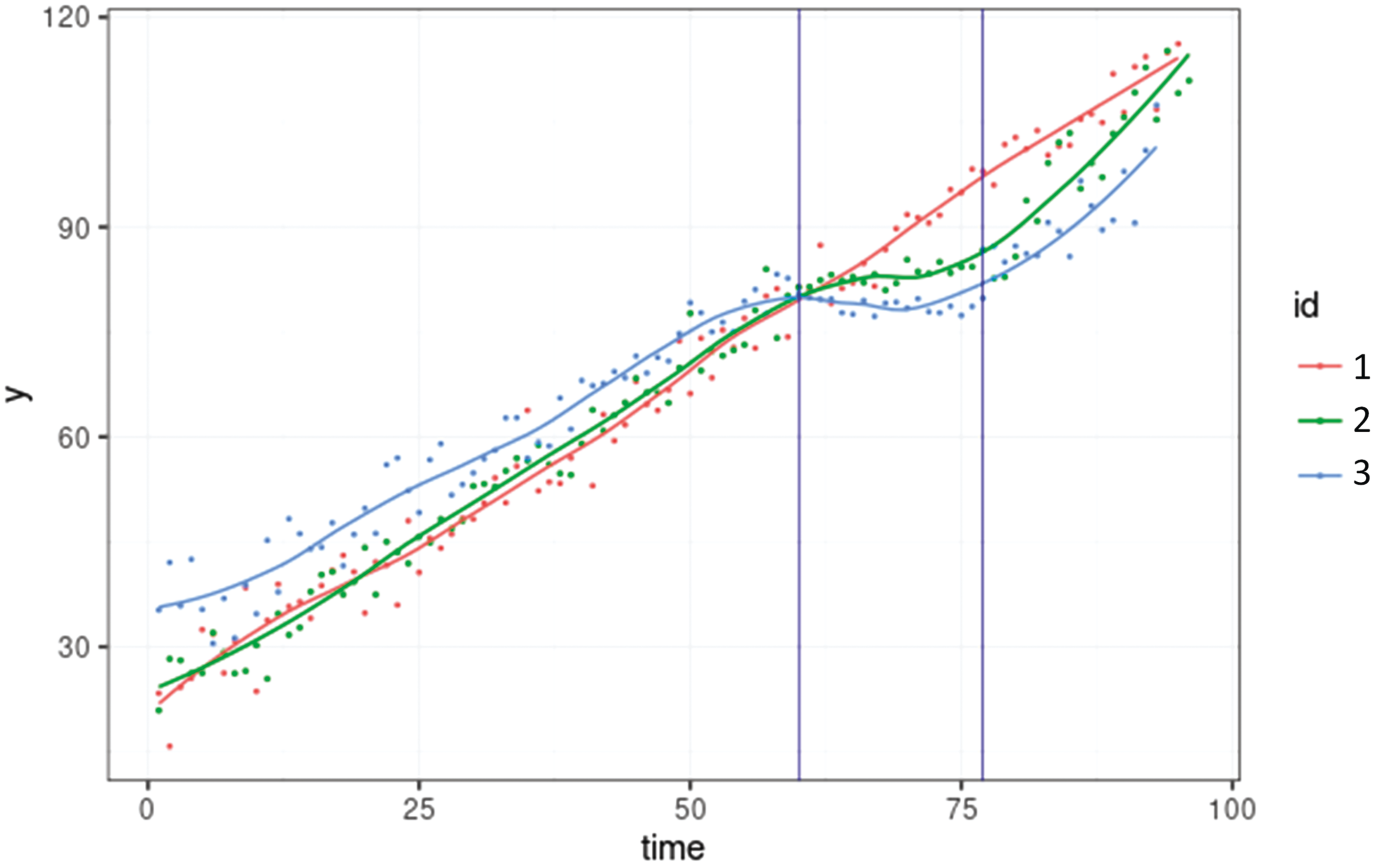
Illustration of the simulated phenotypes (points) associated with their smoothing curve for three animals. Animal 1 did not face any disturbance; animals 2 (resistance = 0.5170, resilience = 0.5490), and 3 (resistance = 0.3650, resilience = 0.5423) belonged to the same pen where a disturbance occurred between days 60 and 77 (vertical lines). The trajectory of animal 3 was more deviate than that of animal 2 due to its lower resistance.

Longitudinal phenotypes of 6,000 individuals were simulated, as described in Le et al. (2022). Following are the main features of these simulations, described in detail in the above-mentioned article. The simulation consists in generating (1) a simplified population of five non-overlapping generations reared in different pens and batches; (2) disturbances of different duration, intensity, and starting date that occur at the individual, pen, or batch level; (3) the resistance and resilience (unobserved traits) of each individual; and (4) the observed longitudinal trait *y*_*ij*_ for each individual *i* at time *j* over a 100-d observation period. For the population simulation, the generation of non-phenotyped founders comprised 12 sires and 150 dams that were randomly mated to give birth to 1,200 offspring (eight offspring per dam, sex ratio = 1/2). Among the progeny, 12 males and 150 females were randomly sampled to be the parents of the next generation. The same process was repeated for each generation. The final population comprised 6,162 individuals. To mimic a production system whereby animals are raised in small groups, animals of G1 to G5 were randomly distributed within each generation across batches and across pens within a batch. Three different types of disturbances were simulated: batch disturbances (all animals in the same batch are subjected to the same disturbance); pen (all animals in the same pen are subjected to the same disturbance); and individual (the disturbance acts on a single animal). The batches, pens, and animals affected by these disturbances were randomly sampled, as were the intensity, starting point, and duration of a given disturbance. The robustness of each animal was modeled using two characteristics: its resistance and its resilience. The resistance on a [0,1] scale, corresponds to the ability of an animal to minimize the direct impact of the disturbance on its performance. A resistance value of 1 indicates an animal insensible to disturbance while a resistance value of 0 corresponds to an absence of resistance, the direct impact of the disturbance will be maximal. Resilience, on a [0,1] scale as well, corresponds to the ability to quickly return to the state before the disturbance. A resilience value of 0 indicates absence of resilience, i.e., the animal will remain in the state it was in at the end of the disturbance. On the other hand, a resilience value of 1 indicates strong resilience, the animal will quickly return to its initial state. Resistance and resilience were considered constant over time for each animal and simulated using a logit genetic model considering a heritability of 0.5 on the underlying scale for both traits. The phenotype’s trajectory over a 100-d period of observation for each individual was simulated using a degree-2 random regression model modified to simulate an elastic response of the animal when exposed to a disturbance. The deviation in trajectory from that which would have been observed in the absence of disturbance is a function of the intensity of the disturbance and the animal’s resistance and resilience. A detailed description of the model is provided in Supplementary Additional File 1. Phenotype trajectories simulated in this way increase over time, with the slope decreasing in the presence of a disturbance, as shown in Figure 2.

Simulations were carried out according to the four-parameter sets described in Table 1. These differed in the percentage of disturbed elements and/or the distribution of the population in batches and pens, and thus aimed at evaluating the impact of the following on the performances of the UpDown method: (1) the percentage of disturbed elements (comparison set 1 vs. 2), (2) disturbance at a lower level or not (set 1 vs. 3), and (3) number of elements in a group level (set 1 vs. 4).

**Table 1.** Sets of parameters used for the simulation

	Set1	Set2	Set3	Set4
#batches per generation	8	8	8	4
#pens within batch	10	10	10	10
#animals per pen	15	15	15	30
% batches disturbed	20	40	20	20
% pens disturbed	20	40	20	20
% animals disturbed	20	40	0	20

Same population for each set: 1,200 phenotyped individuals per generation, five generations.

Using the output of the UpDown method, a genetic analysis of the robustness statistics (Smin, Sresil, and ABT) obtained for animals identified as facing a disturbance occurring at the group level (batch or pen) was performed using animal models by restricted maximum likelihood method using the Average Information algorithm (AI-REML) from the ASReml software (Gilmour et al., 2015). After selection, the fixed effects included in the models were the estimated intensity for all traits and the estimated age when the disturbance started, and the estimated duration of the disturbance for Sresil and ABT. The correlation between the EBV of the different traits and the true BV for resistance and resilience were then calculated. The number of replicates used for the simulation were 1,000 for set 1 and 250 for sets 2 to 4.

### Real data

Data from 5,872 pigs (3,146 Large White [LW], 1,460 Landrace, and 1,266 Pietrain) of the INRAE-France Génétique Porc facility (UEPR, INRAE, Rennes pig experimental unit, France, 2018) in Le Rheu, France, from the year 2017 to 2019, were used in this study. Pigs were raised during the fattening period of around 100 d (from approximately 63 to approximately 162 d of age) in pens equipped with an automatic concentrate feeder (ACF) associated with an animal weighing scale. For each visit to the ACF, individual feed intake, time spent in the ACF and BW were recorded. The animals were distributed into 52 batches and 440 different pens, with no mixing of breeds per pen; there was a median of 9 pens per batch (range [6 to 9]) and 14 individuals per pen (range [9 to 15]). During this period, health data were also available. These data provided information about the treatments received by the studied pigs (animal ID, treatment date, animal main symptom, type of treatment [antibiotic, vaccine, etc.]). In total, 4,919 different pigs received at least one treatment. Based on the type of treatment (curative or preventive) and the proportion of animals in a pen or in a batch receiving the same treatment at the same time, the health data made it possible to identify disturbances occurring at the batch, pen, or individual levels. In addition, the maximal daily outdoor temperature (T in °C) and humidity (H in %) data of the facility location were collected from the ‘Météo-France’ website. The temperature and humidity index (THI) was calculated for each day using the following formula (Wegner et al., 2014): THI=(1.8T+32)−(55H/10,000)×(1.8T−26). Days with a THI greater than or equal to 75 were considered as disturbed days that impacted all batches of animals in the phenotyping station on these days. A total of 30 potential disturbances (25 different batches) at the batch scale (14 due to health problems and 16 associated with high THI), 10 potential disturbances at the pen scale (9 pens) and 1,094 potential individual disturbances (812 animals) were identified.

Detection and characterization of disturbances using the UpDown package and evaluation of Smin, Sresil, and ABT for animals exposed to a detected disturbance were carried out from this database using three different daily phenotypes: CFI, BW, and feed rate (FR). The phenotypes were corrected beforehand by the median trajectory over age (over day for CFI) per breed for each phenotype and divided by their daily standard deviation in order to put phenotypes on the same scale. Heritability of each of the new traits describing the robustness of the animal (Smin, Sresil, and ABT) were then estimated for LW pigs in a multitrait animal model using ASReml (Gilmour et al., 2015). For this analysis, we considered that regardless of the original phenotype used to obtain the robustness criteria (i.e., CFI, BW, or FR), they correspond to the same traits. Only the robustness traits of animals considered to be undergoing a disturbance at group level (and not an individual disturbance) were taken into account for this analysis. Fixed effects included in the models were the batch, the estimated intensity of the disturbance, its estimated duration, and the age of the animal when the disturbance started. The random permanent environmental effect was not included in the models due to its low variance which was not significantly different from 0. In addition, we estimated the genetic relationship between each of the robustness criteria and the phenotypes from which they are derived to evaluate the link between robustness and production. To do so, production phenotypes must reflect the genetic potential for production only and not a combination of production and robustness (Le et al., 2022). Therefore, for animals identified as experiencing disturbances, only phenotypic records registered 4 d before the estimated starting date of the earliest disturbance were used for the analysis. For animals identified as not experiencing any disturbance, phenotypic records registered before the average estimated starting date of the detected disturbances were used for the analysis to limit the difference in the period studied for the two groups of animals. The repeated BW, FI, and FR conserved phenotypes were then summarized per animal in average daily gain (ADG), average daily feed intake (ADFI), and average feed rate (AFR). The genetic correlations between robustness traits and the genetic potential for production, considering that summarized production phenotypes may correspond to different traits depending on whether the animal experienced a disturbance or not, were then estimated using a series of three-trait multitrait animal models (one robustness trait, one summarized production trait for disturbed, and non-disturbed animals). In addition to the additive genetic effects, effects included in the models for the summarized production phenotypes were the age of the animal at the date used to censor the data, and the batch.

## Results

### Simulated data

**Figure 3.**
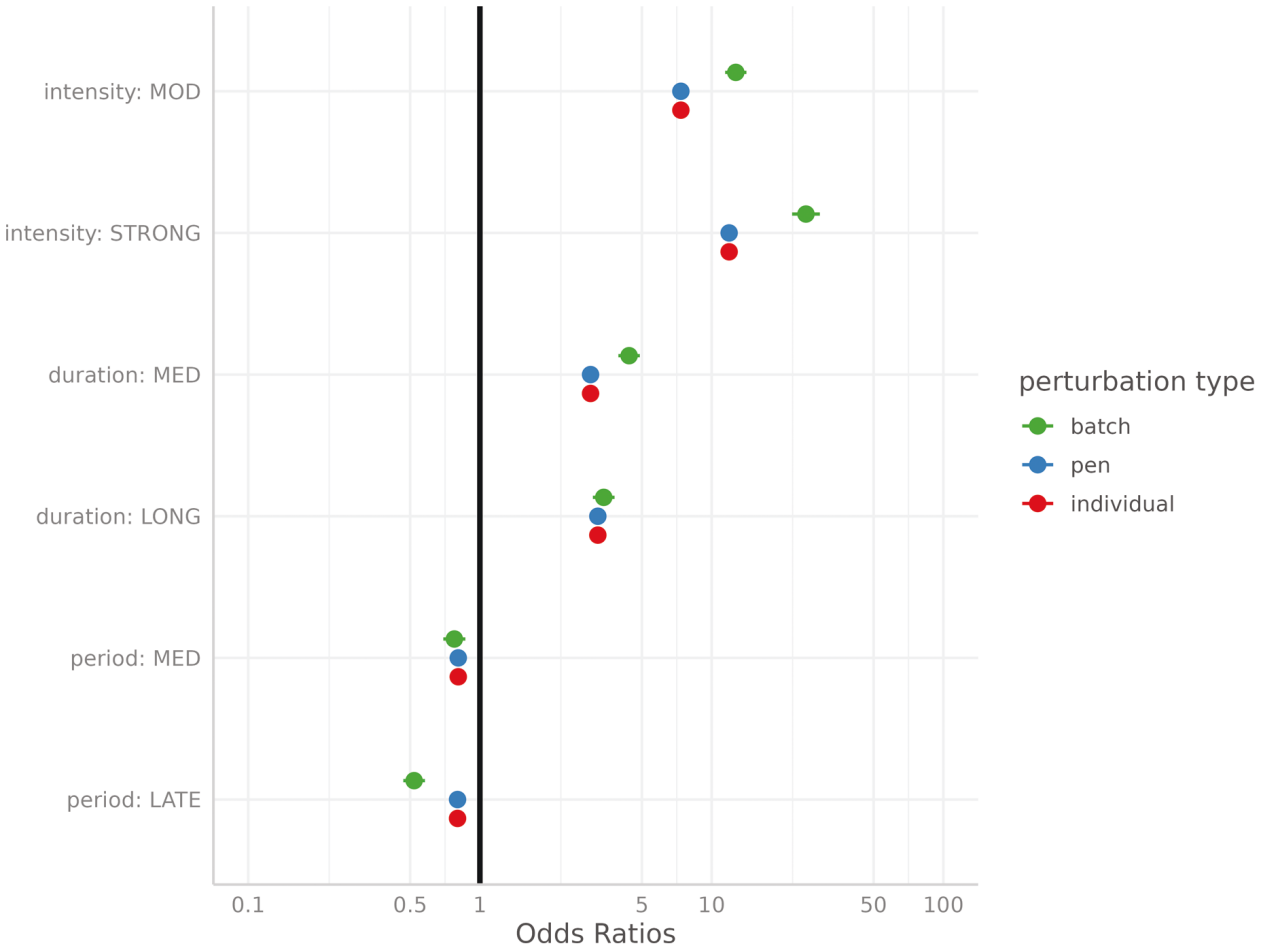
Forest plot of the factors impacting the sensitivity of disturbance detection. Intensity: intensity of the disturbance in three classes, low (<0.7), moderate (MOD; [0.7,1.4]), and strong (≥1.4). Duration: duration of the disturbance in three classes, short (<8d), medium (MED; [8d,16d]), and long (≥16d). Period: period where the disturbance occurs in three classes, early (before day 33), medium (MED) (between days 33 and 66), and late (after day 66).

**Figure 4.**
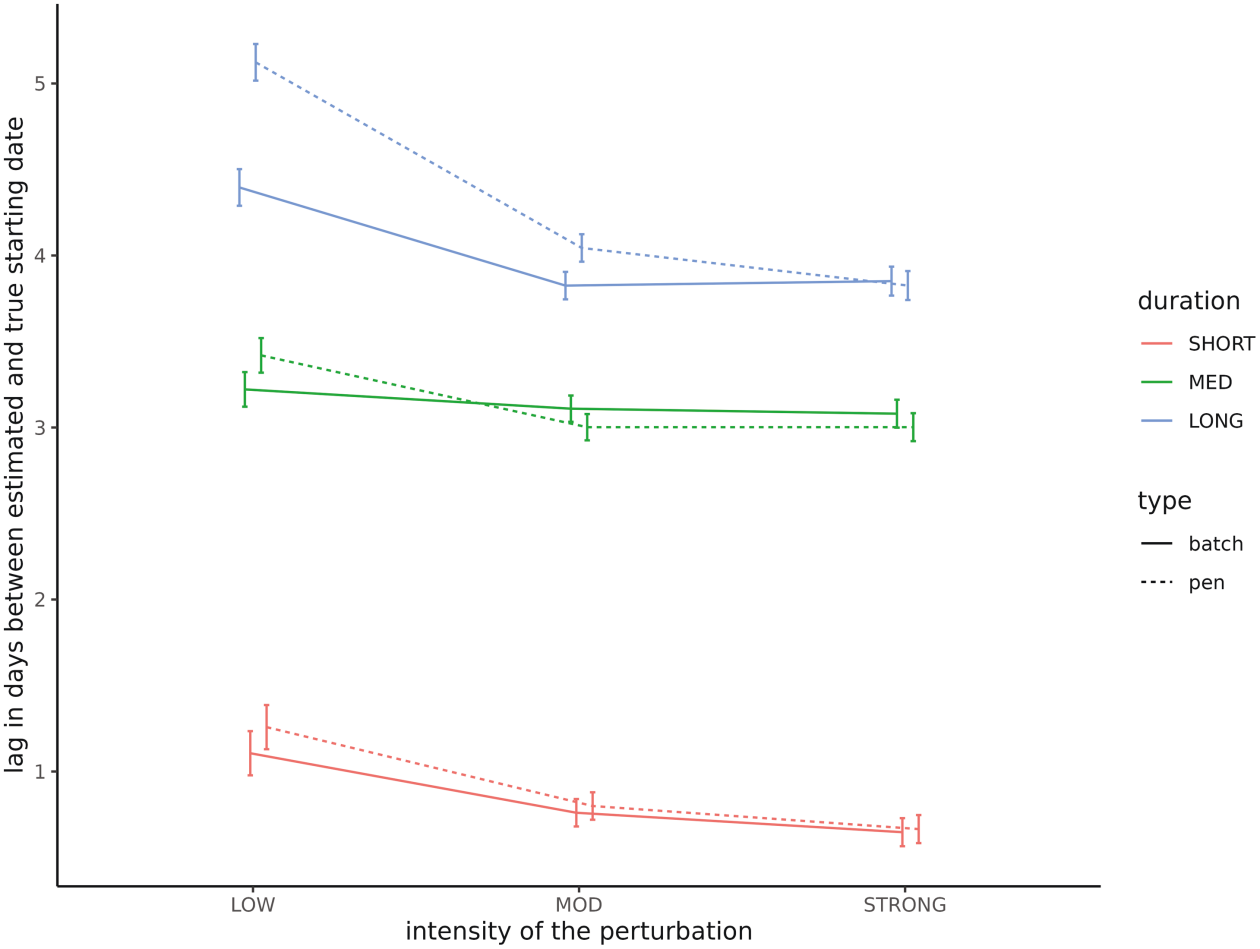
Impact of disturbance duration and intensity on the estimation of its start date. Intensity: intensity of the disturbance in three classes, low (<0.7), moderate (MOD; [0.7,1.4]), and strong (≥1.4). Duration: duration of the disturbance in three classes, short (<8 d), medium (MED; [8d,16d]), and long (≥16 d).

**Figure 5.**
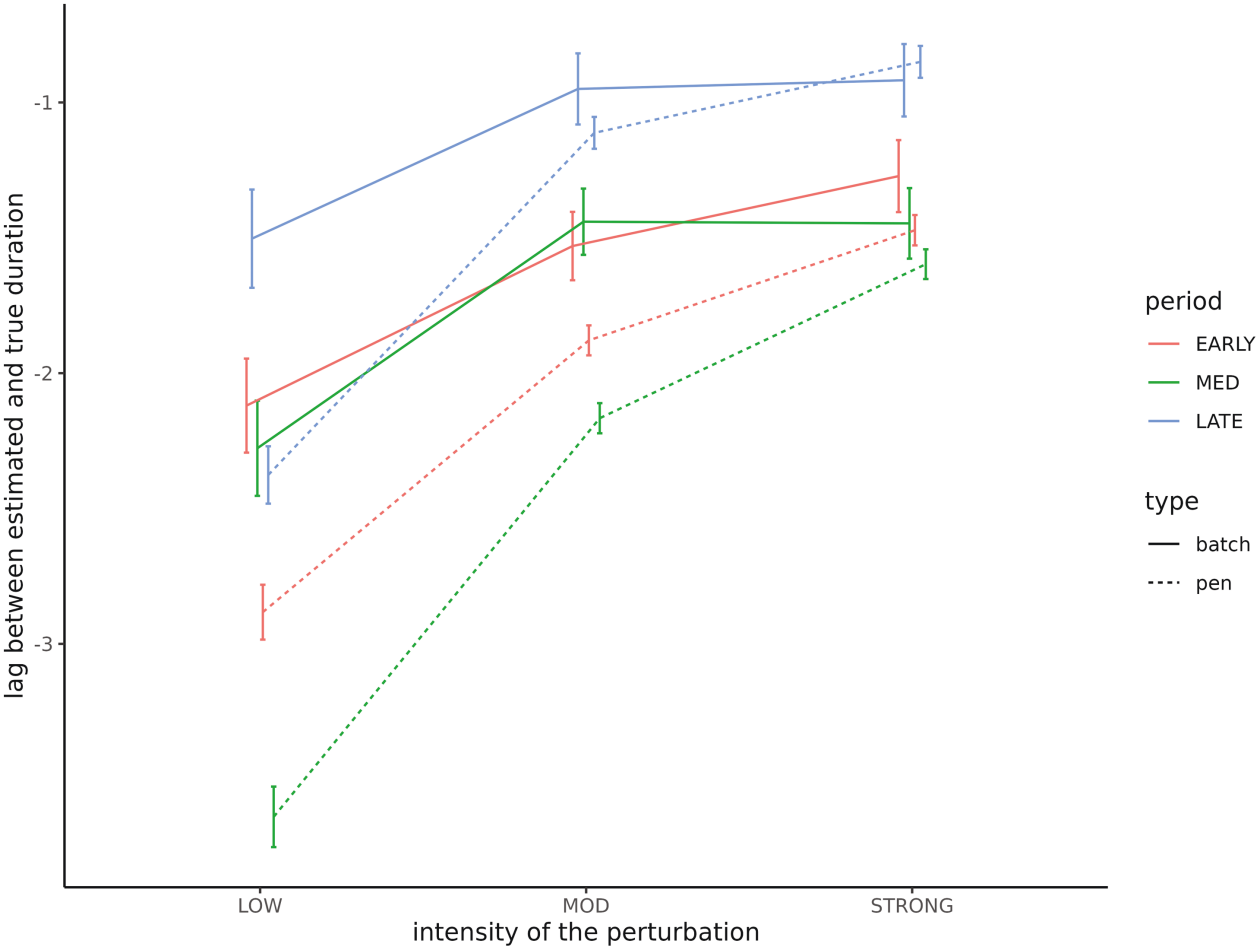
Impact of disturbance intensity and occurrence period on the estimation of its duration. Intensity: intensity of the disturbance in three classes, low (<0.7), moderate (MOD; [0.7,1.4]), and strong (≥1.4). Period: period where the disturbance occurs in three classes, early (before day 33), medium (MED) (between days 33 and 66), and late (after day 66).

**Figure 6.**
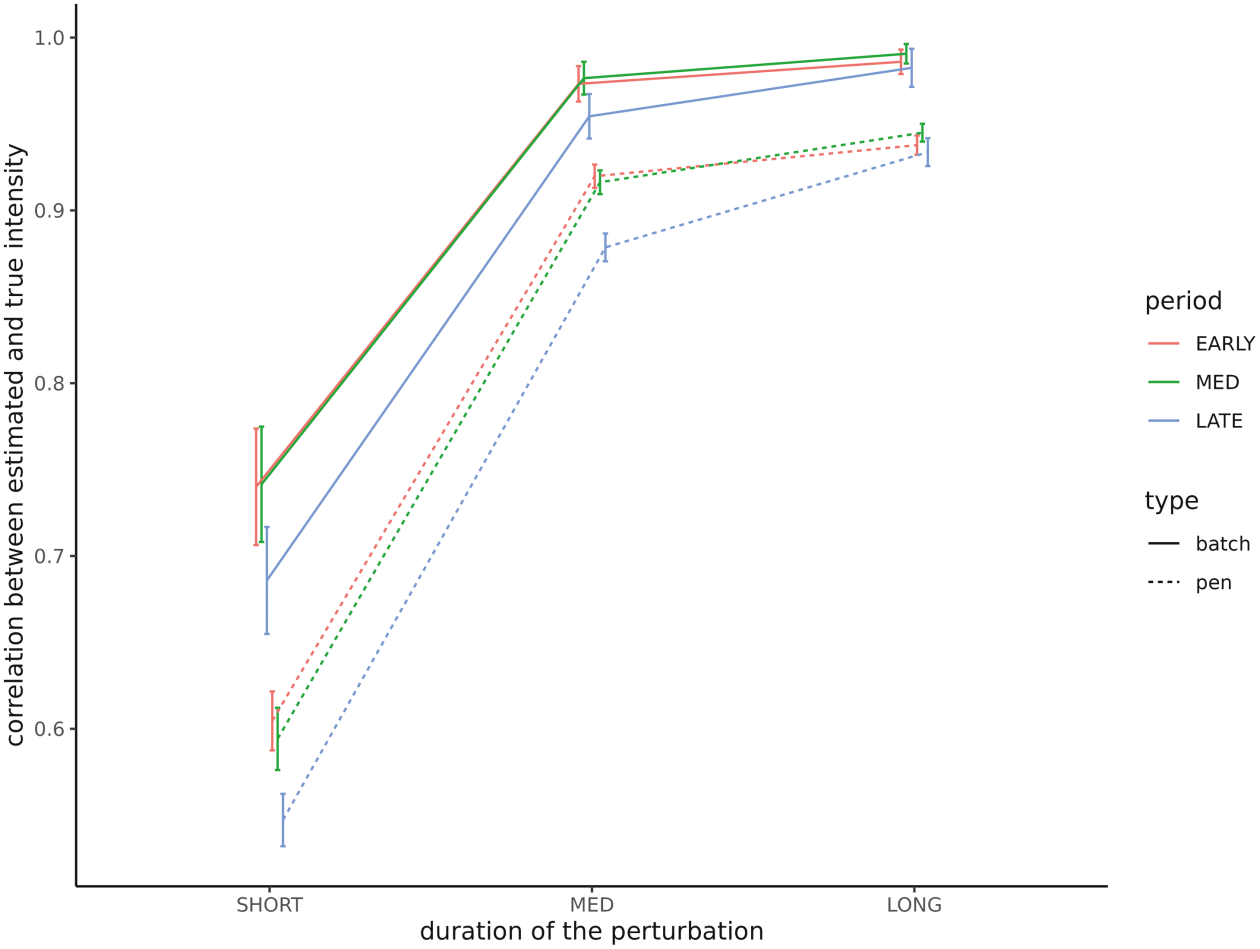
Impact of disturbance duration and period of occurrence of the estimation of its intensity. Duration: duration of the disturbance in three classes, short (<8 d), medium (MED; [8d,16d]), and long (≥16 d). Period: period where the disturbance occurs in three classes, early (before day 33), medium (MED) (between days 33 and 66), and late (after day 66).

The sensitivity (the ability to correctly identify disturbed elements) and the specificity (the ability to correctly identify non-disturbed elements) of the UpDown method for each group level and set of simulations are provided in Table 2. The sensitivity of disturbance detection at the individual level was low, ranging from 0.23 to 0.32 depending on the set of parameters. It increased for higher levels, varying between 0.45 and 0.59 for pen disturbances, and between 0.77 and 0.88 for batch disturbances, the latter being associated with the largest standard errors. We did not observe any statistically significant differences in sensitivity depending on the set of parameters. However, it can be noted that it tended to be lower for all hierarchical levels as the prevalence of disturbance increases (set 1 vs. 2). The specificity of the UpDown method was high regardless of the level of disturbance or the set of parameters ranging from 0.95 to 0.99. We evaluated the impact of the type of disturbance on the sensitivity of detection using a logistic regression model for each level of disturbance separately. Fixed effects included in the models were, in addition to the set of parameters, the intensity, the duration, and the period of occurrence of the disturbance considered as cross-classified factors in three classes each: intensity—low (<0.7), moderate (0.7,1.4), and strong (≥1.4); duration—short (<8 d), medium (8,16 d), and long (≥16 d); and period—early (before day 33), medium (between days 33 and 66), and late (after day 66). Odds ratio estimates of the models are provided in Figure 3. Results were similar regardless of the level at which the disturbance occurred. The main factor affecting the sensitivity of detection was the intensity of the disturbance: the stronger the intensity is, the higher the probability of detection will be. We observed a decrease in probability of detection when the disturbance is of short duration. Finally, disturbances occurring at the beginning of the control period had a slightly higher probability of detection, whereas it was lower for the batch scale when the disturbance occurred at the end of the control period. The quality of the characterization of the detected disturbances depending on the level and the set of parameters is provided in Table 3. The statistics were in the same range for the different sets of parameters. The estimated start date of the disturbance tended to be posterior to the true start date. The average lags between the estimated and true start date were approximately 2.6 and approximately 2.8 d for disturbances occurring at the batch and pen level, respectively, while it was equal to approximately 1.9 d for individual disturbances but associated with a large standard error (approximately 15). The duration of the perturbation tended to be underestimated for the different levels of disturbance in a similar manner (by approximately 1.6 d). The correlation between the true and estimated intensity of the disturbance increased with the hierarchical level of organization (on average, approximately 0.41, 0.78, and 0.83 for individual, pen, and batch disturbances, respectively). The impact of intensity, duration, and period of occurrence of the disturbance on the different statistics was evaluated using linear models. The effect of the intensity and duration of the disturbance on the lag between the estimated and true start date is provided in Figure 4. The longer the disturbance is, the greater the time interval between the estimated and true start dates will be. We also observed a slight increase in this lag when the disturbance is of low intensity. The lag between the estimated and true duration depending on the intensity and period of occurrence of the disturbance is provided in Figure 5. The underestimation of the disturbance duration was greater for disturbances of low intensity. The later the disturbance occurred, the lower the underestimation of its duration was. The impact of the period and the duration of the perturbation on the correlation between true and estimated intensity is represented in Figure 6 for the batch and pen levels. We observed a strong decrease in the correlation when the disturbance was of short duration and a slight decrease when the disturbance occurred later in the control period.

**Table 2.** Proportion of well-detected elements among the disturbed elements (sensitivity) and among the undisturbed elements (specificity) using the UpDown method for the different group levels and sets of parameters (mean ± SD)

Set	Batch	Pen	Individual
	*Sensitivity*		
Set 1	0.82 ± 0.15	0.54 ± 0.07	0.32 ± 0.04
Set 2	0.77 ± 0.13	0.45 ± 0.07	0.23 ± 0.06
Set 3	0.83 ± 0.14	0.55 ± 0.07	—
Set 4	0.88 ± 0.17	0.59 ± 0.10	0.30 ± 0.05
	*Specificity*		
Set 1	0.99 ± 0.02	0.98 ± 0.01	0.95 ± 0.01
Set 2	0.98 ± 0.03	0.98 ± 0.02	0.96 ± 0.01
Set 3	0.99 ± 0.02	0.99 ± 0.01	0.95 ± 0.01
Set 4	0.99 ± 0.04	0.99 ± 0.02	0.95 ± 0.01

Set 1, 20% of all hierarchical levels (batch, pen, and individual) exposed to disturbances; Set 2, 40% of all hierarchical levels exposed to disturbances; Set 3, 20% of batch and pen levels exposed to disturbance. Set 4, 20% of all hierarchical levels exposed to disturbances, four batches.

**Table 3.** Performances of the UpDown method to characterize detected disturbances for the different group levels and sets of parameters (mean ± SD)

Set	Level	Estimated-true start date	Estimated-true duration	Correlation between estimated and true intensity
1	Batch	2.60 ± 1.72	−1.35 ± 3.15	0.83 ± 0.008
	Pen	2.83 ± 4.88	−1.71 ± 3.57	0.77 ± 0.003
	Individual	1.78 ± 14.47	−1.68 ± 5.15	0.42 ± 0.002
2	Batch	2.81 ± 2.79	−1.55 ± 3.15	0.81 ± 0.008
	Pen	2.93 ± 6.30	−1.74 ± 3.85	0.75 ± 0.004
	Individual	1.94 ± 15.29	−1.63 ± 5.49	0.37 ± 0.002
3	Batch	2.54 ± 2.46	−1.31 ± 3.03	0.84 ± 0.009
	Pen	2.72 ± 4.82	−1.69 ± 3.48	0.78 ± 0.004
4	Batch	2.59 ± 1.98	−1.38 ± 3.04	0.83 ± 0.014
	Pen	2.78 ± 3.93	−1.65 ± 3.45	0.81 ± 0.006
	Individual	1.85 ± 14.56	−1.71 ± 5.14	0.43 ± 0.002

Set 1, 20% of all hierarchical levels (batch, pen, individual) exposed to disturbances, Set 2, 40% of all hierarchical levels exposed to disturbances, Set 3, 20% of batch and pen level exposed to disturbance. Set 4, 20% of all hierarchical levels exposed to disturbances, four batches.

Only the robustness criteria of animals having been identified as facing a disturbance occurring at the group level (batch or pen) were used for the genetic analysis. Therefore, only a small part of the animals in the population had phenotypes for these criteria. The proportion of animals with at least one robustness phenotype was on average 39%, 68%, 47%, and 22% for sets 1 to 4, respectively. The results of the genetic analysis of Smin, Sresil, and ABT are provided in Table 4. Heritability of the three traits ranged from low to moderate, depending on the set of parameters: from 0.12 to 0.17 for Smin, from 0.18 to 0.23 for Sresil, and from 0.13 to 0.16 for ABT. The correlation between the EBV of Sresil and the BV for resistance was low regardless of the set of parameters, ranging from −0.11 to −0.14. They were higher for the other two robustness traits, with values ranging from 0.29 to 0.33 for Smin and from 0.16 to 0.21 (in absolute value) for ABT. The correlations between EBV and BV for resilience were low for all traits: lower than 0.05 (in absolute value) for Smin and ABT and varying between 0.09 and 0.11 for Sresil.

**Table 4.** Heritability of the different robustness criteria and accuracy of their EBV for resistance and resilience (mean ρ ± SD)

Set	*h* ^2^	ρ (*EBV*, *u*_*resis*_)	ρ (*EBV*,*u*_*resil*_)
		Smin	
Set 1	0.12 ± 0.14	0.29 ± 0.09	0.05 ± 0.10
Set 2	0.12 ± 0.09	0.33 ± 0.09	0.04 ± 0.08
Set 3	0.17 ± 0.17	0.30 ± 0.09	0.04 ± 0.09
Set 4	0.16 ± 0.17	0.27 ± 0.12	0.03 ± 0.09
		*Sresil*	
Set 1	0.19 ± 0.24	−0.11 ± 0.09	0.09 ± 0.08
Set 2	0.19 ± 0.16	−0.14 ± 0.08	0.11 ± 0.08
Set 3	0.23 ± 0.26	−0.12 ± 0.09	0.10 ± 0.09
Set 4	0.18 ± 0.19	−0.11 ± 0.09	0.09 ± 0.09
		*ABT*	
Set 1	0.13 ± 0.17	−0.17 ± 0.09	−0.03 ± 0.09
Set 2	0.13 ± 0.14	−0.21 ± 0.09	−0.04 ± 0.08
Set 3	0.16 ± 0.20	−0.18 ± 0.09	−0.03 ± 0.09
Set 4	0.13 ± 0.17	−0.16 ± 0.11	−0.03 ± 0.09

Smin, slope minimum; Sresil, slope for resilience; ABT, area-between trajectories.

Set 1, 20% of all hierarchical levels (batch, pen, individual) exposed to disturbances; Set 2, 40% of all hierarchical levels exposed to disturbances; Set 3, 20% of batch and pen level exposed to disturbance. Set 4, 20% of all hierarchical levels exposed to disturbances, four batches.

### Real data

**Figure 7.**
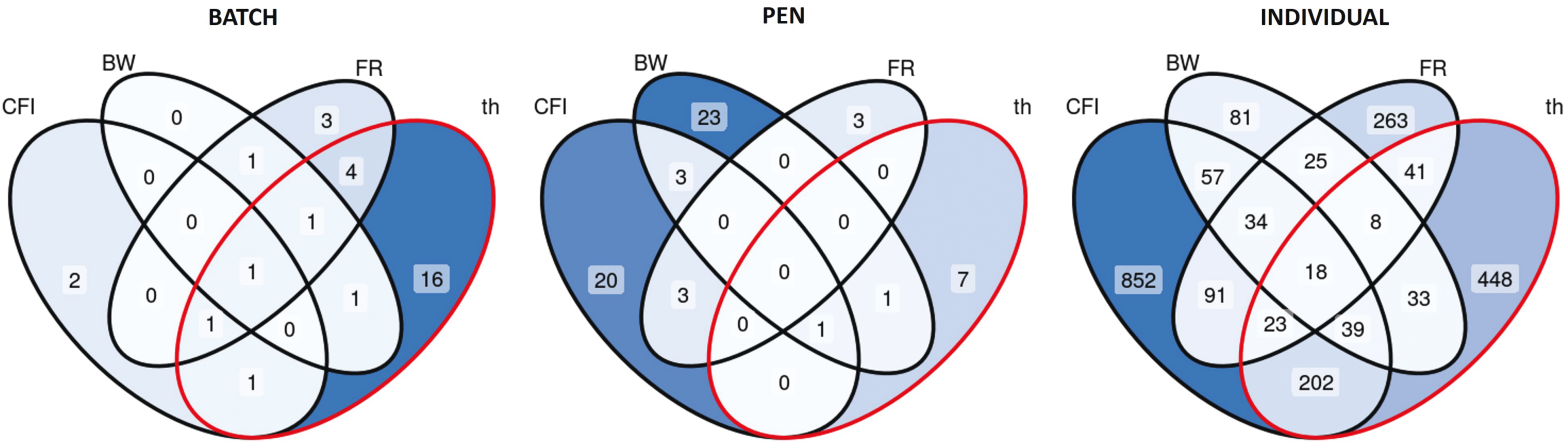
Venn diagram of the elements detected at the different hierarchical levels depending on the phenotype used. BW, body weight; CFI, cumulative feed intake; FR, feeding rate; th, theoretical disturbed elements based on THI and medical treatments.

Applied to real data, the UpDown method identified different elements perturbed depending on the phenotype used. The corresponding Venn diagram is provided in Figure 7. A total of 15 batches were considered as facing a disturbance (5 detected by CFI analysis, 4 by BW, and 11 by FR). Only four batches were detected by multiple phenotype analyses (one in common for the three phenotypes, one in common for FR and CFI and two in common between BW and FR). In addition, nine of the 15 batches detected using the UpDown package were also considered to be disturbed on the basis of meteorological and health data. A total of 54 pens were detected as facing a disturbance (27 CFI, 28 BW, and 6 FR), four in common between CFI and BW and three in common between CFI and FR. Two of the detected pens also faced identified health events. Finally, a total of 1,767 individuals were considered as experiencing an individual disturbance, 52 of them were detected by the three phenotype analyses, 96 by CFI and BW, 114 by CFI and FR, and 33 by FR and BW, resulting in 83% of the individual disturbances detected by one phenotype analysis only. A total of 21% of the detected individuals were also considered as experiencing a sanitary issue based on the treatment data. Robustness criteria of LW pigs detected as experiencing a group disturbance (batch or pen) were used for genetic analysis. Consequently, only 34% (1,171 animals) of the LW pigs have at least one measure of robustness. The heritability, phenotypic, and genetic correlation of Smin, Sresil, ABT, and summarized production traits are provided in Table 5. Heritability of the robustness traits were moderate: 0.20 ± 0.04, 0.16 ± 0.04, and 0.11 ± 0.04 for Smin, Sresil, and ABT, respectively. Sresil was phenotypically and genetically independent from the two other criteria, while Smin and ABT were favorably correlated (−0.76 ± 0.14 for the genetic correlation). The heritability estimates for the summarized production phenotypes were large, varying between 0.37 and 0.55 depending on the phenotype. The phenotypic correlation between summarized production phenotypes and robustness criteria was low (<0.18). The genetic correlation between the summarized phenotype recorded on all animals was high for ADG (0.86 ± 0.18) and ADFI (0.91 ± 0.10) but moderate for AFR (0.60 ± 0.14). All the genetic correlation estimates between Sresil and the summarized phenotypes were low (all ≤ 0.14 in absolute value). The genetic correlation estimates between Smin and ADFI and AFR from non-disturbed animals were low (≤0.08) and slightly positive with ADG (0.25), but associated with a large standard error (0.22). When summarized production phenotypes were derived from the trajectory of animals experiencing a disturbance, the genetic correlations between Smin and production tended to be negative and significantly different from 0 for ADFI and AFR. It should be noted that genetic correlation estimates were generally associated with large standard errors. The ABT criterion was genetically independent of ADFI and AFR recorded on disturbed animals, whereas it was negatively correlated with ADF and positively with AFR recorded on animals not experiencing a disturbance. Finally, we observed a trend of favorable genetic correlation between ABT and ADG (−0.47 and −0.39 for phenotypes of non-disturbed and disturbed animals, respectively), but standard errors of the correlation estimates were large (>0.20).

**Table 5.** Heritability (on the diagonal), phenotypic correlation (above the diagonal) and genetic correlation (below the diagonal) of the different robustness criteria measured on LW pigs

	Smin	Sresil	ABT	ADG_nd_	ADG_d_	ADFI_nd_	ADFI_d_	AFR_nd_	AFR_d_
Smin	0.20 ± 0.04	−0.10 ± 0.03	−0.52 ± 0.03		−0.12 ± 0.03		0.18 ± 0.03		0.09 ± 0.03
Sresil	−0.01 ± 0.17	0.16 ± 0.04	0.07 ± 0.03		−0.09 ± 0.04		0.07 ± 0.04		0.10 ± 0.04
ABT	−0.76 ± 0.14	0.19 ± 0.21	0.11 ± 0.04		0.03 ± 0.04		−0.03 ± 0.04		−0.02 ± 0.04
ADG_nd_	0.25 ± 0.22	0.04 ± 0.26	−0.47 ± 0.30	0.38 ± 0.07	—	0.80 ± 0.02		0.42 ± 0.02	
ADG_d_	−0.17 ± 0.13	0.00 ± 0.16	−0.39 ± 0.22	0.86 ± 0.18	0.39 ± 0.12		0.45 ± 0.03		0.36 ± 0.03
ADFI_nd_	0.08 ± 0.19	0.14 ± 0.21	−0.30 ± 0.24			0.55 ± 0.07	—	0.57 ± 0.02	
ADFI_d_	−0.33 ± 0.10	0.01 ± 0.12	0.01 ± 0.17			0.91 ± 0.10	0.37 ± 0.09		0.81 ± 0.02
AFR_nd_	0.02 ± 0.18	−0.14 ± 0.23	0.17 ± 0.28					0.50 ± 0.07	—
AFR_d_	−0.58 ± 0.08	−0.02 ± 0.13	0.07 ± 0.17					0.60 ± 0.14	0.50 ± 0.09

Smin, slope minimum; Sresil, slope for resilience; ABT, area-between trajectories; ADG, average daily gain; ADFI, average daily feed intake; AFR, average feeding rate. Subscript nd, non-disturbed; d, disturbed.

## Discussion

An extensive literature on the quantification of animal robustness by modeling its environmental sensitivity has emerged in recent years, thanks, in particular, to the development of high-throughput phenotyping tools that offer the possibility to characterize the dynamics of phenotype change in the face of variations in the environment. Under the assumption that animals are permanently subject to micro-variations in their environment, the fluctuation pattern of the phenotype is expected to be informative about robustness (Scheffer et al., 2018; Berghof et al., 2019). These fluctuations are usually summarized into three different statistics derived from the difference between the observed phenotype and its modeling (i.e., residuals): the variance, autocorrelation, and skewness of the residuals (Poppe et al., 2020; Homma et al., 2021; Bedere et al., 2022; Wang et al., 2022). The main difficulty in applying this method is the modeling of the theoretical trajectory of the phenotype, which must not be affected by the disturbances. This poses no problem in the case of micro-variations in the environment but is less obvious in the presence of long-term disturbances (Codrea et al., 2011; Poppe et al., 2020). This is why some authors have sought to identify and characterize disturbances before assessing the animal’s response to these environmental changes. In the context of precision livestock farming, different methods have been used in livestock to identify animals whose longitudinal phenotype deviates from their expectation (Cornou et al., 2008; Maselyne et al., 2018). However, as the evolution of an individual trajectory depends on the disturbance and the robustness of the animal, such animal-by-animal approaches do not allow us to identify the disturbances suffered by the most robust animals, which will not react to the disturbances. Working at the scale of a group of animals avoids this problem, provided that the distribution of animals within a group is independent of their robustness. The UpDown method used in this study was developed around the idea (working at the group scale to detect macro variations), and although individual disturbances are identified and characterized by the method, they are not used to measure animal robustness for the reason mentioned above. It is very important to bear in mind that the UpDown method is designed to identify and measure deviations in trajectories corresponding to a response to a disturbance over the relatively long term (macro variation) and does not pretend to capture micro variation, which is correctly assessed by the residual analyses. The idea is that these long-term deviations are generally erased by the modeling of the animal’s individual curve (in random regression model for instance) and therefore do not impact the residuals. The criterion currently used in the method to distinguish disturbed from non-disturbed elements is the minimum slope of the trajectory. The underlying hypothesis behind this choice is that disturbance always goes in the same direction (slowing down or accelerating evolutionary dynamics over time). Other criteria were also tested during the development of the UpDown method, derived from the analysis of the residuals (variance, skewness, and autocorrelation) or from the study of the group variance over time (Garcia-Baccino et al., 2021), but resulted in poorer performance in terms of the sensitivity and specificity of disturbance detection (Le, 2022). The increase in sensitivity of the UpDown method to detect disturbances from the individual to the batch scale confirms that summarizing trajectories at the group scale level helps to detect disturbances by removing micro-variations (whether noise or not) in the studied trajectories that constitute the group. We can assume that sensitivity would have been further increased at a level above that of the batch, e.g., the breeding region. However, it must be borne in mind that the number of elements at each level must be sufficiently large to allow the mixture model to converge. The characterization of group disturbances enabled by the method can also be used as a posteriori evaluation tool on the farm, helping to identify stress factors and levers for improvement.

Even if not statistically significant, we observed that the sensitivity of detection of the UpDown method tended to decrease with the prevalence of the disturbances (comparison set 1 vs. 2). This surprising result, given that no effect of the prevalence had been anticipated, is probably the consequence of the precorrection of the data for the general trend of the population over age. The precorrection aims at removing any natural trajectory pattern over time. In this study, this involved subtracting the median value of the phenotype for the corresponding age in the population from the observed phenotype. In set 2, several by-age median values may be affected by the disturbances since a proportion of animals exceeding 50% may be exposed to a disturbance at the same age. This leads to a higher variance of the distribution of the minimal slopes of disturbed elements in the mixture model and, thus, a greater difficulty in separating the disturbed from the non-disturbed elements. However, a situation where 40% of the batches, the pens, and the individuals are exposed to a disturbance of non-negligible intensity is an extreme case that may not often be observed in practice, especially in breeding farms. The lack of difference in sensitivity and specificity of the UpDown method for detecting pen perturbations between sets 1 and 3 (sub-elements disturbed or not) shows the effectiveness of the method in eliminating perturbations of underlying scale elements by using the median phenotype value for a given scale and by validating the detected element in the Down-step of the method. It is important to note, however, that this would not have been the case if, by misfortune, more than 50% of the underlying elements were exposed to different perturbations, but at the same time (however, this “for validation proportion” can be modified in the UpDown package parameters). We did not highlight a significant difference in pen disturbance detection depending on the number of individuals in a pen (15 or 30 individuals, set 1 vs. 4), probably because the number of individuals remains sufficiently large in the two situations to avoid confusion between individual and pen disturbances. The extreme case of only two individuals in a pen would not have made it possible to separate the two types of disturbances (pen and individual). This is why, when we applied the UpDown method to the real pig data, we kept animals of three breeds to detect disturbances in order to avoid batches with a very small number of pens with records. As expected, the main characteristic of the disturbance influencing the sensitivity of detection was its intensity, where a disturbance of low intensity induced changes in the phenotype that were too small to be detected. A disturbance of short duration will also be less likely to be detected since the UpDown method smooths the data, eliminating deviations of excessively short duration. It should be noted that, if necessary, the smoothing parameter can be modified in the package to adapt the method to the nature of the data being analyzed. We observed that a late disturbance is less likely to be detected, especially at the batch scale. The reason is probably due to the fact that not all animals have phenotype records at the end of the test period because recording stops at 100 d of control (to mimic departure to the slaughterhouse). Consequently, for the last days of observation, the number of animals with records per pen and thus per batch were too small to permit disturbance detection by the UpDown method.

Once a disturbance is detected, the UpDown method extracts its characteristics: end and start dates, and intensity. The estimated disturbance start date is almost always slightly later than the actual date (for more than 94% of the cases for pen and batch disturbances and more than 83% of the cases for individual disturbances). The lag between the true and estimated start date was generally lower at the individual scale. This result does not mean that the start date of individual disturbances is better estimated than the start date of group level disturbances. This reduction in average lag between true and estimated start date is the consequence of trajectories identified as modified over a given period, which, in fact, do not correspond to the periods when the disturbances actually occurred, leading to a large gap between the true and estimated start dates. Indeed, for around 10% of the disturbances occurring at the individual level, the difference between the estimated and true start date (absolute value) was greater than 10 d, while this percentage was <1% for the disturbances occurring at the pen or batch scale. This is also reflected in the large standard deviation associated with the average gap between the start and end of the disturbance identified at the individual scale, while the median value of this gap was similar for all disturbance scales. The tendency to underestimate the duration of the disturbance is directly linked to different estimation biases for disturbance start and end dates. Both are too late estimated but the end date of the disturbance is generally more accurately estimated than the start date (<1 d/true end date). The later estimate of the disturbance start date (and thus underestimation of the duration) was probably the consequence of trajectory smoothing (the minimal value of its first derivative being used to localize the start of the disturbance), which is all the more important the longer the disturbance duration and the lower the disturbance intensity are. When the disturbance occurs late, the duration of the disturbance is less underestimated since these disturbances are generally shorter (9.9 d.) than those occurring earlier (12.7 d.) since the data is censored at 100 d (duration of the test period). The main factor affecting the estimation of the intensity of the disturbance is its duration. We noted that in the case of short duration, the intensity of disturbances of high true intensity tended to be underestimated. Because the smoothed trajectory cannot fall abruptly and rise rapidly over a short period, the smoothed trajectory will have a smoother evolution than the underlying noiseless trajectory in the event of a short disturbance and, therefore, a lower downward slope. To sum up, the main performance shortcomings of perturbation characterization using the UpDown method are linked to data smoothing. The default value of the bandwidth for the Nadaraya-Watson’s smoothing used in the UpDown package, tuned to maximize sensitivity and specificity of the method based on our simulated data, is *n*^1/4^ where *n* is the number of time points. This value can be modified by the user (the R shiny App ‘UpDownApp’ provided in the package UpDown can help in the choice of the best smoothing parameter). However, a bandwidth value that is too small will lead to high oscillations of the smoothing curve and, thus, false detection of disturbed elements (i.e., a reduction in the method’s specificity), whereas, on the other hand, a value that is too high will lead to an over-smoothing and the algorithm might not detect the disturbances (i.e., reduction of the sensitivity).

The three criteria: Smin, Sresil, and ABT, extracted from the analysis of the trajectories of animals undergoing a detected disturbance, should reflect the resistance, resilience, and a combination of both (robustness). Their estimated heritability were low to moderate, far from the 0.50 values used to simulate the underlying resistance and resilience potentials because these heritabilities are not comparable. Indeed, resistance and resilience are concepts that are not directly measurable. They are only expressed when the animal is exposed to a disturbance through a change in the trajectory of an observed phenotype. The variability of this change is linked to the environmental variability of the production phenotype itself and to the variance of the smoothing curve (Tsybakov, 2009). In other words, the residual variance of the production phenotype also contributes to those of the measured robustness criteria. To illustrate our point, we carried out 50 additional simulations of set 3 with very low residual variance for the production phenotype (results not shown) leading to noise in the trajectory of the same order of magnitude as that observed for CFI and BW in the real data. Heritability estimates were then higher for Smin and Sresil: 0.23 and 0.43 on average, respectively; but similar for ABT (0.17). In addition, the proposed criteria are only proxies of these changes in trajectory. Furthermore, only animals detected as experiencing a group-level disturbance have measured phenotypes. Nonetheless, given the non-negligible correlation between their EBV and BV for resistance, the two criteria, Smin and ABT, can provide an indication of the genetic potential of the animal in terms of resistance. Based on the simulation results, Sresil was the phenotype that gave the higher accuracy for resilience and thus remains the best proxy for this trait. Nonetheless, the accuracy was low. This may be because it is necessary to estimate the animal’s end-of-response date to determine this slope, which is not feasible when the disturbance occurs late. Furthermore, this estimated slope depends on resilience as well as on the difference that exists at each time point between the observed phenotype and the value it should have had in the absence of disturbance, which may tend to add noise to the measurement.

When applying the UpDown method to real data, we standardized the longitudinal phenotypes (BW, CFI, and FR) in order to obtain disturbance characteristics and Smin, Sresil, and ABT on the same scale, regardless of the longitudinal phenotype used. The aim of this is to make it possible to analyze the criteria as the same trait and thus increase the number of animals with Smin, Sresil, or ABT records to avoid bias in the heritability estimates. Nonetheless, this pretreatment of the data may have slightly reduced the sensitivity of the UpDown method. The UpDown method detected different disturbances depending on the phenotype used. This is not surprising since the resource acquisition and allocation strategies (Friggens et al., 2017) that animals implement in response to disturbance may depend on the type of disturbance and the animal (Ben Abdelkrim et al., 2021). For instance, in response to heat stress, animals can reduce their feed intake, feeding rate, and/or their activity, which, in turn, have or do not have consequences on their ADG (Gourdine et al., 2019; Mayorga et al., 2019). Nevertheless, we would have hoped for more concordance between analyses of different phenotypes. This would perhaps have been the case if the data analyzed had been from commercial farms where the environment is less well controlled (Lenoir et al., 2022) and where disturbances of high intensity, with consequences on multiple phenotypes, can be expected. In the experimental farm considered in this study where the environment is highly controlled, so that the disturbances encountered were a priori of low intensity, the impact of disturbances on multiple phenotypes is less obvious. In the same vein, the concordance with the disturbances suggested by the analysis of treatment and climate data was low as well. When a treatment takes place in such facilities, it is usually early before the spread of the disease and the animals have therefore not had the time to be highly exposed (more preventive than curative treatment). This seems to be confirmed by the results obtained. Indeed, 54% of the batches that received a curative treatment were identified as disturbed by the UpDown method, but at least one pen of each of the five undetected batches receiving a treatment has been identified as disturbed (1, 4, 2, 1, and 2 pens of these five batches, respectively). In addition, at least one animal of each of the seven pens considered as experiencing a sanitary challenge based on treatment data but not detected as being a disturbed pen using the UpDown method, was detected by the UpDown method as facing an individual disturbance. The temperature and humidity data used in the study were measured outside the facility and may not accurately reflect the environmental conditions inside the facility. That may explain why only three (21%) of the batches considered as having suffered thermal stress based on climate data were also detected by the UpDown method.

Genetic parameters were estimated considering that robustness criteria correspond to the same trait regardless of the longitudinal phenotype used to obtain it. We tried to investigate this hypothesis by performing additional analysis. We estimated the genetic correlation for each robustness criterion, considering the trait as different according to the type of longitudinal phenotype used to obtain it (CFI, BW, and FR), through a series of two-trait models. It was difficult to get all the models to converge, and estimates of genetic correlations were associated with large standard errors, thus results should be interpreted with caution. The genetic correlation between robustness traits obtained with BW or CFI were >0.9 for the three robustness criteria, and varied between 0.40 and 0.71 between those obtained with FR and those obtained with the other phenotypes, depending on the criterion considered. It would therefore seem that the robustness traits obtained with BW and CFI correspond to the same trait, although this is less obvious for those obtained with FR. Heritabilities obtained for the three criteria were low to moderate, and tended to fall within the highest heritability values reported in the literature for different robustness/resilience proxies. The heritabilities based on the study of the residuals were reported to be generally low (<0.15 [Berghof et al., 2019]), but recent studies have reported moderate values of heritability for the logarithm of the residual variance (≃0.20 [Poppe et al., 2020]) and RMSE (≃0.25 [Putz et al., 2019]). The heritability of the area-between-curves criterion proposed by Revilla et al. (2022), which corresponds more or less to our ABT criterion, was low (0.03 to 0.04), as were the robustness scores proposed by Lenoir et al. (2022) based on routinely recorded phenotypes at the end of the growing period in pig (≃0.09). Provided that Smin was, as anticipated, a proxy of the resistance and Sresil a proxy of the resilience, we found no genetic correlation between these two components of robustness. To the best of our knowledge, there is no study in the literature that has estimated this genetic correlation. The heritabilities obtained on the real data were greater than those obtained on the simulated data, probably because the trajectories of the real production phenotypes were less noisy than the simulated ones (illustration of real BW and CFI in Supplementary Additional File 2).

In order to evaluate the relationship between robustness and production, we used summarized production phenotypes derived from undisturbed longitudinal phenotypes, i.e., recorded before any disturbance was detected. The summarized phenotypes must correspond to the same criterion regardless of whether the animals are exposed to a disturbance or not and must reflect the true production potential of the animals. This seems to be the case for ADG and ADFI for which the genetic correlations between the two situations (disturbed or not) were high, but not for AFR for which the correlation was only 0.60. A slightly weaker correlation for a trait may be due to the fact that: (1) the sensitivity of UpDown based on this trait is lower than expected; (2) the start date of the disturbance estimated by UpDown on this trait is much later than the true start date. Consequently, the data used to calculate summarized phenotype comprised disturbed observations; and/or (3) the trait considerably changes with age. It is not possible to evaluate the true sensitivity of UpDown based on FR. Nevertheless, we suspect that the sensitivity of pen disturbance detection using FR was low, since the number of pens detected by FR is almost five times lower than that detected by CFI or BW. This may be linked to the quality of the measurement, which is probably less accurate than that of ADG or FI because it accumulates the uncertainties of the different variables used to calculate it: FI and time spent at the ASF per visit (in addition an animal can remain in the ASF without eating resulting in an error in the calculation of FR). For disturbances detected using FR and another production phenotype, the start dates of disturbance estimated using FR were generally earlier (3.4 d on average) than those estimated using the other phenotypes, which does not support the second hypothesis but is consistent with the intuition that FR reacts early to disturbance. Multiple trait animal models applied to the by-period (four distinct periods of 25 d each: [1,25], [26,50], [51,75], and [76,100]) summarized phenotypes of animals not detected as exposed to any disturbance did not provide any arguments in favor of the third hypothesis, the average genetic correlation between successive periods being in the same range for ADG (0.85), AFI (0.91) than AFR (0.94). The phenotypic correlation between the summarized production phenotypes were in line with those reported by Rauw et al. (2006), strong correlation between ADG and FI, and positive correlation between FR and the other production traits. It is difficult to draw conclusions from genetic correlation estimates between robustness criteria and production because of their large standard errors. It is nevertheless interesting to note the trend towards very low genetic correlations between Smin and production traits, which would indicate, provided that Smin is a good indicator of the animal’s resilience, the independence between resilience and production. On the other hand, the positive correlation between Smin and ADG in the non-disturbed situation (0.25), and the negative correlation between ABT and ADG indicate a favorable genetic correlation between resistance, robustness, and growth, which is not consistent with the allocation theory (Friggens et al., 2017). In any case, further investigations on larger or different datasets are needed to confirm or refute these trends.

Given that the heritability of ABT was the lowest, integrating the Smin and Sresil traits into the selection objective seems to be the most appropriate approach for improving robustness. In any case, before considering it, it is still necessary to validate them as good proxies for resistance and resilience, especially for Sresil for which the results of the simulation were not favorable. A way to validate them could be to evaluate the genetic correlation between these traits and functional traits such as longevity, but that are difficult to identify in the pig production system where culling is rapid. However, longevity corresponds to robustness over a long-term period of time, whereas the resistance and resilience that we have considered correspond to short-term responses, and it is unclear how long and short-term robustness are related (Friggens et al., 2022). The best option would be to perform two divergent selections, one on Sresil and the second on Smin, and to evaluate the dynamic response of the descendent in the face of a well-controlled challenge.

## Conclusion

This study presented a validation and an application of the UpDown method to detect and characterize disturbances and to estimate robustness of animals via three proxies. The results presented throughout this study proved the relevance of the UpDown method to reach these goals. To further evaluate the interest of this method for characterizing disturbances, it would be interesting to apply it to other real datasets under less controlled conditions and/or known/provoked disturbances and/or with other population structures. The three proposed robustness criteria are promising, but still need to be validated.

## Supplementary Material

skae059_suppl_Supplementary_File_S1

skae059_suppl_Supplementary_File_S2

## Abbreviations

ABT: area-between trajectories
ACF: automatic concentrate feeder
ADFI: average daily feed intake
ADG: average daily gain
AFR: average feed rate
BW: body weight
CFI: cumulative feed intake
FR: feed rate
LW: large white
Smin: slope minimum
Sresil: slope for resilience
th: theoretical disturbed elements
THI: temperature humidity index
