# Supplementary material for: Identification and characterization of unknown disturbances in a structured population using high-throughput phenotyping data and measurement of robustness: application to growing pigs
Source: J Anim Sci. 2024 Mar 5;102:skae059. doi: 10.1093/jas/skae059 (PMC10977036; doi:10.1093/jas/skae059)
Supplement: skae059_suppl_Supplementary_File_S1 [file skae059_suppl_supplementary_file_s1.docx]

**Additional file 1: models used to simulated phenotype**

Phenotype $y_{ij}$ of animal $i$ at time $j$ was simulated as:

$$y_{i1}=16+{age}_{i1}+\sum_{k=0}^{2} a_{k,i}\varphi_{k}\left( {age}_{i1} \right)+e_{i1}$$

$$y_{ij}=y_{i\left( j-1 \right)}^{*}+\Delta_{ij}\left[ 1+A_{ij}-B_{ij} \right]+e_{ij} ,\forall j>1$$

where ${age}_{ij}$ is the age of the animal at day $j$, $y_{i\left( j-1 \right)}^{*}=y_{i(j-1)}-e_{i(j-1)}$, $\Delta_{ij}=1+\sum_{k=0}^{2} a_{k,i}\left[ \varphi_{k}\left( {age}_{ij} \right)-\varphi_{k}\left( {age}_{i\left( j-1 \right)} \right) \right]$ it corresponds to the expected change (i.e in the absence of disturbance) in phenotype for animal $i$ from time $\left( j-1 \right)$ to $j$. $A_{ij}={1+resil}_{i}\frac{z_{ij}}{y_{i1}}\left( 1-\frac{y_{i\left( j-1 \right)}^{*}}{z_{i\left( j-1 \right)}} \right)$ where $z_{ij}$ corresponds to the expected phenotype of animal $i$ at time $j$ if it has never faced disturbance, i.e $z_{ij}=15+j+{enter\_age}_{i}+\sum_{k=0}^{2} a_{k,i}\varphi_{k}\left( {age}_{ij} \right)$, and ${resil}_{i}={logit}^{-1}\left( u_{resil,i}+e_{resil,i} \right)$ is the resilience of animal $i$, $B_{ij}=\left( 1-{resis}_{i} \right) {int}_{ij}$ where ${resis}_{i}={logit}^{-1}\left( u_{resis,i}+e_{resis,i} \right)$ is the resistance of animal $i$ and ${int}_{ij}$ is the sum of the intensities of the disturbances that animal $i$ is facing at time $j$. $\mathbf{a}_{\mathbf{k}}\boldsymbol{,}\boldsymbol{u}_{\boldsymbol{resis}}\boldsymbol{,}\boldsymbol{u}_{\boldsymbol{resil}}$ are the additive genetic effects with distribution

$$\left[ \begin{matrix} \boldsymbol{a}_{\boldsymbol{0}} \\ \boldsymbol{a}_{\boldsymbol{1}} \\ \begin{matrix} \boldsymbol{a}_{\boldsymbol{2}} \\ \boldsymbol{u}_{\boldsymbol{r}} \\ \boldsymbol{u}_{\boldsymbol{R}} \end{matrix} \end{matrix} \right]\sim\boldsymbol{N}\left( \boldsymbol{0,}\mathbf{G}\otimes\mathbf{A} \right)\boldsymbol{,}\mathrm{with} \mathbf{G}\boldsymbol{=}\left[ \begin{matrix} 4 & 0.62 & -0.36 & 0 & 0 \\ & 2.4 & -0.28 & 0 & 0 \\ & & 0.8 & 0 & 0 \\ & sym & & 5 & 0 \\ & & & & 5 \end{matrix} \right]\boldsymbol{,}$$

where $\mathbf{A}$ is the genetic relationship matrix. Residuals $e_{ij}$ were independent and had centered Gaussian distributions with variance at day $j$ equal to ${{11 \varphi}_{0}\left( j \right)}^{2}+{8\varphi_{1}\left( j \right)}^{2}+0.6{\varphi_{2}\left( j \right)}^{2}$, $\mathbf{e}_{\mathbf{resis}}\sim N\left( 0,5\mathbf{I} \right)$ and $\mathbf{e}_{\mathbf{resil}}\sim N\left( 0,5\mathbf{I} \right)$.

The start dates of the disturbance were uniformly selected between day 1 and 99. The durations of the disturbances were uniformly sampled between 1 and 25 days. Finally, the intensities of the disturbances were sampled according to the uniform distribution on (0.5,2.5). In the simulations, the animals can undergo at most one disturbance of each kind, but disturbances at different levels are not exclusive. Therefore, an animal may be exposed throughout its life to a batch disturbance, a pen disturbance and an individual disturbance that may occur at the same time.
