# Supplementary material for: Identification and characterization of unknown disturbances in a structured population using high-throughput phenotyping data and measurement of robustness: application to growing pigs
Source: J Anim Sci. 2024 Mar 5;102:skae059. doi: 10.1093/jas/skae059 (PMC10977036; doi:10.1093/jas/skae059)
Supplement: skae059_suppl_Supplementary_File_S2 [file skae059_suppl_supplementary_file_s2.docx]

**Additional file 2 : Cumulative feed intake and body weight of a growing pig during the fattening period sampled from the real dataset**


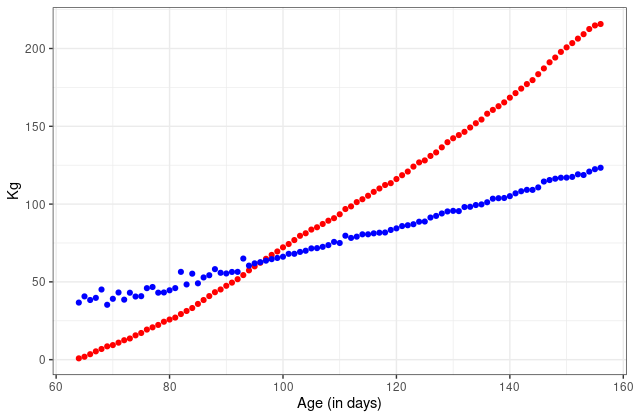


Red points :Cumulative feed intake, blue points : Animal body weight. This animal has been identified as experiencing a batch disturbance between d6 (69d. of age) and d26.
